# Glycogen Synthase Kinase-3 (GSK-3) Regulation of Inhibitory Coreceptor Expression in T-cell Immunity

**DOI:** 10.33696/immunology.3.115

**Published:** 2021

**Authors:** Mark E. Issa, Christopher E. Rudd

**Affiliations:** 1 Department of Medicine, Université de Montréal, Montreal, Quebec, Canada; 2 Division of Immunology-Oncology, Centre de Recherche-Hospital Maisonneuve Rosemont, Montreal, Quebec, Canada H1T 2M4

## Abstract

The serine/threonine kinase, glycogen synthase kinase 3 (GSK-3) has been implicated in immune cell activation and function. Our recent studies have shown that the abrogation of GSK-3 activity down-regulates the expression of key inhibitory receptors PD-1 and LAG-3. It also regulates the expression of the transcription factor NFAT which, in turn, is responsible for inhibiting PD-1/LAG-3 transcription as well as activating the expression of cytolytic effector proteins such as perforin and granzyme B. The role of components of the Wnt signaling pathway in these events remains to be fully uncovered. This mini-review discusses the recent discoveries that have elucidated the role of the GSK-3 signaling pathway in cancer immunotherapy.

## Introduction

While the immune system is capable of recognizing tumor antigens, certain cancer cells evade immune detection and destruction [1]. Due to genetic instability, cancer cells become heterogeneous with an ever-accumulating mutational burden that can render the immune system exhausted and incapable of dealing with the expanding tumor. In addition to the genetic instability, cancer cells utilize a variety of mechanisms that enable them to evade immune destruction and promote tumor escape [2]. The tumor microenvironment can create conditions that limit the immune response by impairing antigen presentation, releasing immunosuppressive cytokines, exhausting the availability of oxygen and nutrients, and promoting the recruitment of immunosuppressive regulatory T-cells (Tregs), neutrophils and myeloid derived suppressor cells (MDSCs) [1,3].

As in the case of classic peptide antigen presentation, tumor neoantigens can be presented by major histocompatibility complexes I and II (MHC-I and MHC-II) leading to T-cell activation [3,4]. This involves the engagement of the receptor (TCR) and coreceptors, CD4 and CD8 which we showed bind to the protein-tyrosine kinase p56^lck^ (LCK) for the induction of a protein tyrosine phosphorylation cascade [5,6]. LCK phosphorylates the tyrosine residues in the immunoreceptor tyrosine-based activation motifs (ITAMs) of the ζ chains of the TCR complex [7–9]. This creates docking sites for second protein tyrosine kinase termed ZAP-70 [10]. ZAP-70 phosphorylates immune cell adaptor proteins, linker of activated T-cells (LAT) and the SH2-domain containing signal transducing adaptor protein (SLP-76) [11,12]. Phosphorylated LAT serves as a docking site for SLP-76, ultimately leading to T-cell activation, proliferation, differentiation, and cytokine release. In general terms, *src* kinases such as p56^lck^ phosphorylate a wide array of substrates, while ZAP-70 is more restricted in its number of targets [5].

**Figure 1:**
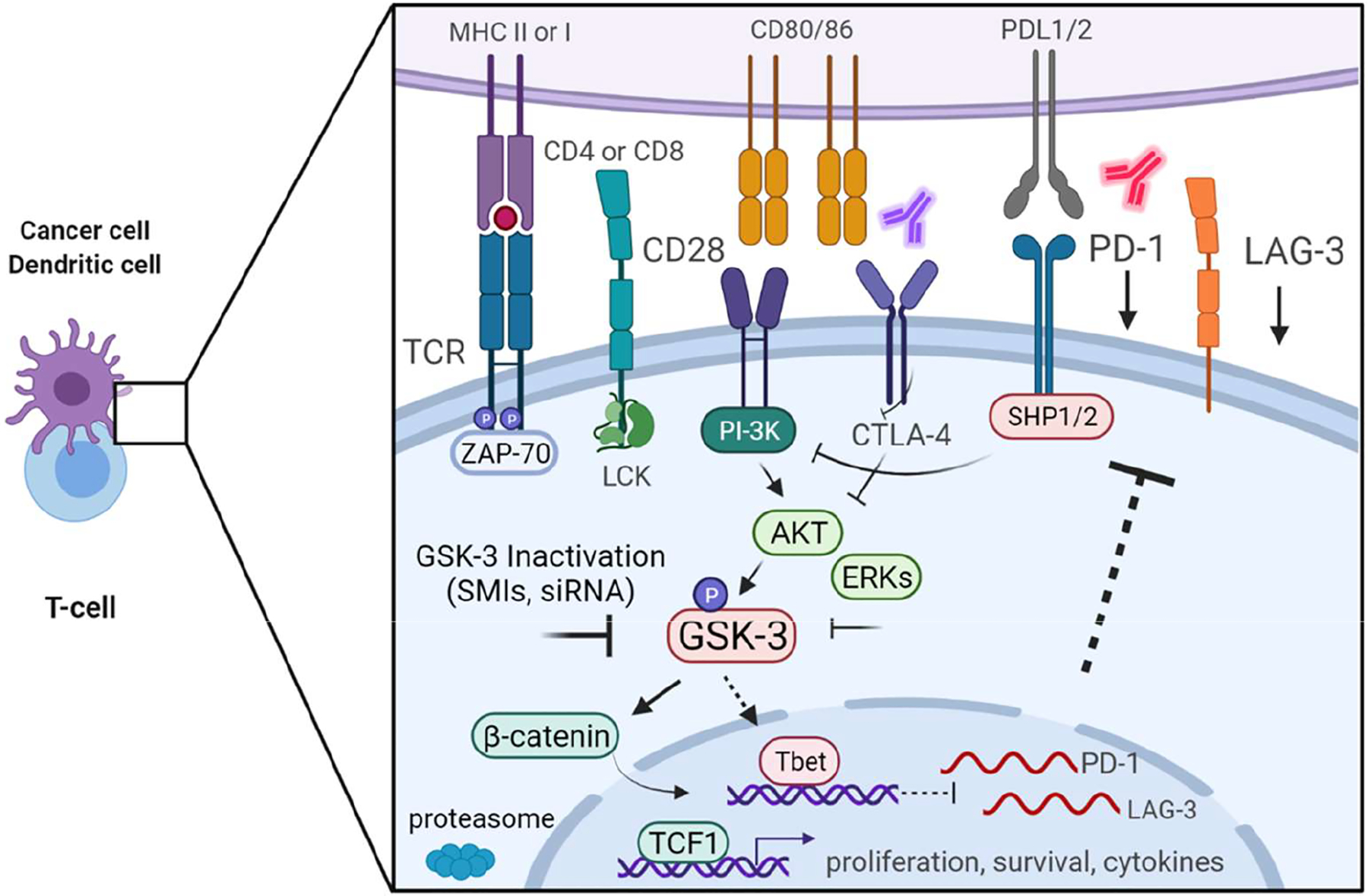
GSK-3 regulates PD-1 and LAG3 expression for enhanced T-cell reactivity. T-cell activation is regulated by IRs PD-1, LAG-3 and CTLA-4. GSK-3 inactivation by GSK3 small molecule inhibitors (SMIs) or siRNA causes the upregulation of *Tbet* (*Tbx21*) transcription and expression, which in turn, represses the transcription and expression of PD-1 and LAG-3. Some inhibition is mediated via TCR-CD28 mediated activation of PI 3K, its activation of AKT (and ERKs) which phospho-inhibit GSK-3; however, the effects of this pathway are partial. The use of GSK-3 SMIs and siRNAs provides a much more potent bolus Inhibition of GSK-3 resulting in the potent generation of PD-1 and LAG-3 down-regulation. The downregulation of both IRs removes the inhibitory effect of the IRs on T-cell function, allowing for more potent reactivity against tumor antigens in cancer immunotherapy. At the same time, it is possible that PD-1 and CTLA-4 themselves can also affect the activation status of GSK-3. PD-1 and CTLA-4 inhibit AKT activation via different mechanisms resulting in enhanced GSK-3 inhibitory functions. Blockade of PD-1 and CTLA-4 with antibodies might block function and sequester the receptors away from CD28 resulting in enhanced AKT, reduced GSK-3 and reduced PD-1 and LAG3 expression. Lastly, the Inhibition of GSK-3 liberates β-catenin from proteasomal degradation and allows it to translocate to the nucleus to upregulate progenitor transcription factor, TCF-1. TCF-1 regulates T-cell exhaustion and progenitor pool. This figure was created by biorender.com [64].

Following TCR activation, CD8^+^ T-cells differentiate into memory, memory-effector and effector cytolytic T-cells, whereas CD4^+^ T-cells differentiate into helper T-cells, or suppressive Tregs [13,14]. Effector T-cells expand, clear pathogens, and undergo apoptosis or differentiate into memory T-cells for extended immune protection against the re-encountered pathogens [15,16]. However, when a chronic condition, such a cancer, persistent Stimulation can impair T-cell activity, rendering them dysfunctional in a process called T-cell exhaustion [17]. This state of the T-cell is characterized by a progressive loss of T-cell effector function. In this context, TCR activation induces the expression of inhibitory receptors (IRs) such as programmed cell death protein 1 (PD-1), lymphocyte activation gene 3 (LAG-3) and cytotoxic T lymphocyte associated protein 4 (CTLA-4) [18,19]. These IRs can mark exhausted T-cells and to varying degrees regulate T-cell exhaustion. In both cancer biology and in chronic models of infection, the blockade of these receptors (aka immune Checkpoint blockade (ICB)) can partially reverse the state of T-cell exhaustion, thereby unleashing T-cells to proliferate and reject cancer or the infectious agent [1,20]. In the hallmark case of cancer, antibodies to CTLA-4 followed by antibodies to PD-1, or in combination, are effective in reducing tumor growth (Figure 1) [1,21].

## PD-1 and T-cells - A Summary

PD-1 is an IR expressed on the surfaces of T-cells, B cells, and NK cells [22]. It belongs to the CD28 supergene family and plays a critical role in the regulation of immunological tolerance, and in immune cell responses against pathogens and cancer [23,24]. PD-1 is comprised of 288 amino acids with an extracellular immunoglobulin (Ig) domain, a transmembrane domain and a cytoplasmic domain [23,24]. Following TCR activation, basal PD-1 is phosphorylated by LCK on the cytoplasmic immunoreceptor tyrosine-based inhibitory motif (ITIM) and on the immunoreceptor tyrosine-based switch motif (ITSM). The phosphorylated PD-1 ITIM and ITSM act as docking sites for phosphatases SHP-1 and SHP-2, respectively [3]. The phosphatase activity of SHP-1 and/or SHP-2 may target ZAP-70 of the TCR and phosphatidylinositol 3-kinase (PI-3K) of CD28, thereby, terminating the TCR-mediated and CD28-mediated T-cell stimulation [25–27]; although this is still a matter of debate, for example, as to whether SHP-2 acts to positively or negatively regulate T-cells [28].

Importantly, PD-1 binds to two ligands, the B7 homologues 1 and 2 (aka PD-L1/PD-L2). PD-L1 and PD-L2 engagement of PD-1 inhibits the proliferation, cytokine production and effector function in both helper CD4^+^ and cytolytic CD8^+^ T-cells [29,30]. Further, PD-1 blockade requires the expression of the positive co-receptor CD28 to mediate its positive effects [31,32]. Early work from the Riley laboratory reported that PD-1 signaling inhibits AKT phosphorylation by preventing CD28-mediated activation of PI-3K (Figure 1) [33]. In contrast, CTLA-4 was found to inhibit AKT (PKB) phosphorylation in a pathway involving the serine phosphatase PP2A. Others have extended this theme by showing that PD-1 targets the RAS-RAF-MEK-ERK pathway by reducing the activation of MEKs1/2 and that of ERKs1/2, possibly by impairing the activity of PLCγ1 and RAS [34,35]. In a similar vein, PD-1 also plays a key role in the regulation of T-cell metabolism, whereby upon activation, T-cells shift from oxidative phosphorylation to glycolysis [36]. CD28 signaling stimulates this glucose uptake and promotes glycolysis [37]. In contrast, PD-1 ligation was found to reduce both glucose transport and glycolysis [38]. Further, inhibition of PI-3K/AKT pathway caused T-cells to shift to lipid metabolism [36], an effect that promotes T-cell memory [39]. The use of anti-PD-1 was found to restore glycolysis in CD8^+^ TILs [40].

## LAG-3 and Its Role in T-cells - A Summary

LAG-3 is another IR that is expressed on activated T-cells, B cells, and NK cells [22]. It consists of 530 amino acids that include four Ig-like sequential domains, a single transmembrane region, and a short cytoplasmic domain. LAG-3, like CD4, binds to MHC-II, through its membranedistal IgG domain extra loop, and negatively regulates CD4^+^ T-cells [41,42]. It can also negatively regulate CD8^+^ T-cells, possibly through interactions with fibrinogen-like protein 1 (FGLl) [43]. However, the negative effects exerted by LAG-3 on T-cells is mediated by an unusual amino acid sequence, KIEELE, located on the cytoplasmic tail [42]. LAG-3 inhibits T-cell function and controls memory T-cell development [44] and is upregulated in response to cytokines IL-2, IL-7 and IL-12 [45].

In addition, LAG-3 has been reported to identify new Treg subpopulations and promote their suppressive function [46]. In this context, Tregs from LAG-3−/− mice exhibit reduced suppressive activity. Further, LAG-3+ Tregs exhibit superior suppressive activity when compared to LAG-3− Tregs, release more immunosuppressive cytokines IL-10 and TGFβl [47]. LAG-3 has also been found to cooperate with PD-1 to regulate both CD4^+^ and CD8^+^ T-cell function [48]. While LAG-3 and PD-1 double knock-out (KO) mice exhibit an elevated autoimmune disease phenotype affecting multiple organs and leading to death, the majority of the same type of mice effectively cleared tumors and showed a significantly prolonged survival [49].

## GSK-3 and Its Role in CD8+ T-cells

Given this background, it is of a great interest that the expression of PD-1 and LAG-3 is controlled by the serine/threonine kinase glycogen synthase kinase 3 (GSK-3) (Figure 1). This kinase was first discovered in 1980 to regulate glycogen synthase and was subsequently found to phosphorylate over 100 different proteins [50]. GSK-3 has been implicated in numerous cell and disease states such as Alzheimer, diabetes, inflammation and cancer [51]. The two GSK-3 isoforms, α and β are 98% identical in their kinase domains. Importantly, unlike other kinases, GSK-3 is constitutively active in resting T-cells where it maintains T-cells in a quiescent state [50]. GSK-3 enters the nucleus of CD4^+^ T-cells to promote the exit of NFAT, while active GSK-3β inhibits the proliferation of T-cells [52,53]. This occurs independently of the action of the GTP exchange factor, VAV-1 [54]. While TCR and CD28 signals inactivate GSK-3 in T-cells [18,54,55], CD28 activation of PI-3K generates lipids phosphatidylinositol-(4,5)-biphosphate (PIP2) to phosphatidylinositol -(3,4,5)-triphosphate (PIP3) that attach to the plasma membrane to recruit proteins with pleckstrin homology (PH) domains. Pyruvate dehydrogenase kinase 1 (PDK1) in combination with an unidentified kinase phosphorylates and activates PH domain carrying PKB or AKT. PKB (and other kinases) inactivate GSK-3β and GSK-3α by phosphorylating inhibitory residues 9 and 21.

In the context of PD-1 and LAG-3, we first showed that GSK-3 plays a central role in the regulation of PD-1 and LAG-3 in T-cells (Figure 1) [56,57]. Together with former lab member, Dr. Alison Taylor (currently at the University of Leeds), we showed that the use of small molecule inhibitors (SMIs) or siRNA to attain GSK-3 inhibition (GSK-3i) led to a significant increase in CD8+ T cytolytic activity. This increase in CD8+ T cytolytic function was initially found to be due to a GSK-3i-mediated reduction in the expression of PD-1 and later LAG-3. We showed that GSK-3 inhibition reduced tumor growth to the same extent as anti-PD-1 therapy in a B16 melanoma and an EL4 lymphoma model of tumor growth [58]. GSK-3i also promoted the clearance of the lymphocytic choriomeningitis virus clone 13 to the same extent as anti-PD-1 [56]. The combination of anti-LAG-3 and GSK-3 inhibitor further reduced tumor growth and in particular, prolonged survival of mice, more than single agent therapy. Mechanistically, the effect of GSK-3 inhibition is mediated, in part, by an increase in the expression of the transcriptional regulator Tbet (*Tbx21*). Tbet, in turn, binds to and inhibits the transcription of PD-1 and LAG-3 [56,57]. GSK-3 inactivation also compensates for the lack of CD28 in the priming of CD8+ cytotoxic T-cells [59]. On a cellular level, GSK-3 inhibition led to a decreased T-cell motility, reduced CD8^+^ T-cell interactions with target cells but without affecting their cytolytic activity [58]. Overall, GSK-3 and its regulation of IR expression has a great potential as a key pathway to be exploited in the development of novel strategies to treat cancer.

## GSK-3 and the Wnt Pathway

Classically, GSK-3 was discovered to phosphorylate the transcriptional regulator β-catenin rendering it a target for proteasomal degradation, and thereby inactivating important Wnt signaling [59]. Ittherefore remains an issue of future work to determine what components (if any) of the Wnt pathway may be involved in the downregulation of IRs. The Wnt pathway could operate in a way to control IR expression or in a parallel pathway that cooperates with the GSK-3-Tbet-IR axis. The Wnt pathway promotes the presence of self-renewing multipotent stem like CD8^+^ memory T-cells (Tscm). Adoptively transferred Tscm cells exhibit an increased *in vivo* recall and an improved tumor control [60]. The β-catenin-Wnt pathway also regulatesthe function of the transcription factor, T-cell factor 1 (TCF-1). TCF-1 helps maintain T-cells in a more progenitor-like self-renewing state [61]. The ablation of TCF-1+ PD-1+ CD8^+^ T-cells significantly attenuates anti-PD-1-induced tumor control. With respect to exhaustion, TCF-1+ progenitor cell subsets gradually lose TCF-1 expression and give rise to an effector-like Tex intermediate and a terminally exhausted subset [62]. TCF-1 and T-bet facilitate the interconversion between different exhausted T-cells subsets. The exact interrelationship between GSK-3 activity, its control of PD-1 and LAG-3 expression and the interconversion of different stages of exhaustion remain to be explored. In a similar context, TCF-1 was found to associate and competes with Foxp3 in human Tregs where GSK-3 inhibitor mediated Wnt activation significantly impaired Foxp3 activity and reduced Treg suppressive function [63]. The development of interventions linked to the inter-relationships between GSK-3, IRs, exhaustion and suppression on immunity has great potential in the future generation of new therapies in cancer immunotherapy.
